# Coupling of Bioreaction and Separation via Novel Thermosensitive Ionic Liquids Applied in the Baker’s Yeast-Catalyzed Reduction of Ethyl 2-oxo-4-phenylbutyrate

**DOI:** 10.3390/molecules25092056

**Published:** 2020-04-28

**Authors:** Yuexi Yang, Yugang Shi, Lifang Feng, Shiyi Tian

**Affiliations:** School of Food Science and Biotechnology, Zhejiang Gongshang University, Hangzhou 310018, China

**Keywords:** thermosensitive ionic liquids–solvent biphasic system, coupled bioreaction and separation, baker’s yeast-catalyzed reduction, ethyl 2-oxo-4-phenylbutyrate, ethyl *(R)*-2-hydroxy-4-phenylbutyrate

## Abstract

The use of baker’s yeast to reduce ethyl 2-oxo-4-phenylbutyrate (EOPB) in conventional biphasic systems is hindered by low productivities due to mass transfer resistance between the biocatalyst and the substrate partitioned into two different phases. To overcome the limitation, a new reaction-separation coupling process (RSCP) was configured in this study, based on the novel thermosensitive ionic liquids (ILs) with polyoxyethylene-tail. The solubility of ILs in common solvents was investigated to configure the unique thermosensitive ionic liquids–solvent biphasic system (TIBS) in which the reduction was performed. [(CH_3_)_2_N(C_2_H_5_)(CH_2_CH_2_O)_2_H][PF_6_] (c_2_) in 1,2-dimethoxyethane possesses the thermosensitive function of homogeneous at lower temperatures and phase separating at higher temperatures. The phase transformation temperature (PTT) of the mixed system of c_2_/1,2-dimethoxyethane (*v/v*, 5:18) was about 33 °C. The bioreaction takes place in a “homogeneous” liquid phase at 30 °C. At the end of each reduction run, the system temperature is increased upon to the PTT, while c_2_ is separated from 1,2-dimethoxyethane with turning the system into two phases. The enantiomeric excesses (e.e.) of ethyl *(R)*-2-hydroxy-4-phenylbutyrate (*(R)*-EHPB) increased about 25~30% and the yield of ethyl-2-hydroxy-4-phenylbutyrate (EHPB) increased 35% in TIBS, compared with the reduction in 1,2-dimethoxyethane. It is expected that the TIBS established in this study could provide many future opportunities in the biocatalysis.

## 1. Introduction

The interest in the reaction-separation coupling process (RSCP) has been growing in green chemistry and chemical engineering [1,2,3,4,5,6], especially in biocatalytic manufacturing processes [7,8,9,10,11]. The RSCP offers an interesting option for equilibrium limited reaction systems by selective removal of one of the products and thereby increasing the product yield [12,13,14,15,16]. It plays an important role in promoting the practical development of biocatalysis. In recent years, the RSCP based on ionic liquids has been used for selective transport of organic compounds [17,18,19,20,21,22].

Ionic liquids (ILs), especially most of the second generation of air- and water-stable ILs, are increasingly utilized as possible environmentally benign alternative reaction media in biocatalysis in place of conventional solvents [23]. However, the inherently high viscosity of ILs may have unfavorable effects caused by the mass transfer resistance on bioreactions [24,25,26,27,28]. Adding organic cosolvents lowers the viscosity of ILs, but cancels many of the advantages of using ILs [29]. Kong et al. found a temperature-sensitive ionic liquid (IL) that can be employed to the biphasic catalysis system (ionic liquid/heptane) possessing the property of “a homogeneous phase under high temperatures, phase separation under low temperatures” [30]. Based on the above findings, the catalyst and the substrate could be in a mono-phase under high temperature to improve reaction efficiency, and the catalyst and the product could be separated and distributed into two different phases, respectively, with the decrease of temperature until a second phase appears. Based on biphasic separation after the monophasic reaction, the problems of contact resistances between catalysts and substrates could be solved. Recently, we have explored a unique novel functionalized ionic liquid, which has been used as a reaction medium to facilitate the enzymatic esterification of FA with lauryl alcohol [31,32]. Moreover, the thermosensitive ILs could also be employed to build a novel biphasic catalysis system to develop to be a new bioreaction-separation coupling technique to improve the efficiency of biotransformation.

It is well noticed that enzyme activity generally depends on the anions of ILs. ILs were then formed with weakly coordinating anions such as BF_4_ and PF_6_ that were more air-stable and water-stable. Moreover, the negative effect of hydrogen-bonding on the enzyme activity in the presence of ILs can be associated with the anions effect and their action as hydrogen-bonding acceptors for the protein (lipases). The Hofmeister ion interaction toward protein stabilization is well known in protein sciences [33]. José L. Iborra et al. reported that enzyme activities clearly depend on the nature of the ions, the results were improving as the alkyl chain length of the imidazolium cation increased, and as a function of the type of anion ([PF_6_], [BF_4_], or [ethylsulphate]) [34]. Of note, is how the increase in the alkyl chain length of imidazolium cation improves the synthetic activity in all derivatives for ILs based on the [PF_6_] anion, in agreement with previous results obtained for the synthesis of citronellyl esters catalyzed by Novozym 435 [35]. However, for ILs based on the [BF_4_] anion, all the immobilized lipases decreased their activity as the alkyl chain of the imidazolium cation increased. Several authors have described [Bmim][PF_6_] as being a more suitable medium than [Bmim][BF_4_] for different synthetic reactions catalyzed by the immobilized or aqueous solution of free lipases [36,37,38]. The low activity displayed by all immobilized lipases in [Emim][MS] agrees with the previously reported effect of most water-miscible ILs to strip essential water molecules from the enzyme microenvironment in anhydrous conditions, producing protein unfolding and deactivation [39,40,41]. In addition, Itoh et al. reported that both the reaction rate and the enantioselectivity of the reaction depended on the anionic part of ILs ([BF_4_], [PF_6_], [SbF_6_], [OTf], and [TFA]) [42]. They found that hydrophobic IL containing [PF_6_] could act as a favorable solvent in the reaction because lipase was anchored by this IL, and remained in it after the extraction workup of the product due to the insolubility of this IL in both water and extraction of the organic solvent. In addition, as an outstanding example, the fine-tuning of ILs has allowed the design of a lipase-friendly solvent that also enables the dissolution of challenging substrates, such as sugars, cellulose, fatty acids, and triglycerides. Being compatible with enzymes, such as ILs containing a quaternary ammonium structure act as ideal media for enzymatically producing derivatives of many substrates. To achieve this, these enzyme-compatible ILs pertain some common structural features: Multiple ethers and/or hydroxyl groups, to optimize the solvent properties (IL viscosity, H-bond basicity, and water affinity) for mild IL-enzyme interactions [43,44,45].

**Scheme 1:**
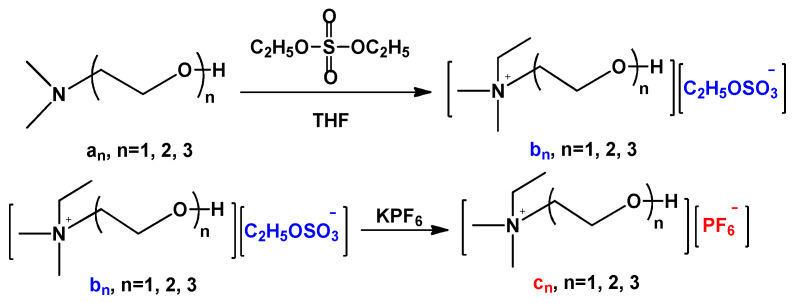
Synthetic routes to ionic liquids (b_n_, c_n_).

Similar to many other whole cell biocatalysis in conventional biphasic systems, the use of baker’s yeast to produce enantiomerically pure ethyl *(R)*-2-hydroxy-4-phenylbutyrate (*(R)*-EHPB) is hindered by low productivities due to mass transfer resistance between the whole cell biocatalyst with the substrate ethyl 2-oxo-4-phenylbutyrate (EOPB) partitioned into two different phases. In this study, the main aim is to design and synthesize novel quaternary ammonium salts with polyoxyethylene-tail type ILs (b_n_, c_n_) (Scheme 1). In addition, the thermosensitive ionic liquids–solvent biphasic system (TIBS) was configured by using these ILs with the intriguing function of “mono-phase under low temperature, two phases under high temperature”. Furthermore, the TIBS was applied to the asymmetric reduction of EOPB with baker’s yeast.

## 2. Results and Discussion

### 2.1. Solubilities of the Ionic Liquids (ILs)

For the construction of a new reaction-separation coupling process (RSCP) with the application of thermosensitive ILs, the primary task is to clear the solubility of these novel ILs in common solvents. As seen from Table 1, the ammonium-based ILs show excellent solubility in strong polar solvents such as water, methanol, and ethanol, while they have poor solubility in nonpolar organic solvents such as nonane, benzene, and petroleum ether at 20 °C. In addition, the solubility of the ILs in 1,2-dimethoxyethane is greatly affected by not only the anion but also the chain length of polyethylene oxide (PEO). As for the ILs with C_2_H_5_SO_3_^−^, only b_1_ can be soluble in 1,2-dimethoxyethane. However, both c_1_ and c_2_ with the hexafluorophosphate anion (PF_6_^−^) are soluble in 1,2-dimethoxyethane. Moreover, b_2_ and c_2_ with the same PEO chain length show the opposite solubility behavior in 1,2-dimethoxyethane, and the same phenomenon can be observed for b_3_ and c_3_ in acetone, suggesting that the anion plays a crucial role in the solubility of ILs.

In order to establish the thermosensitive ionic liquids–solvent biphasic system (TIBS), the solubility of the ILs with various chain lengths of polyethylene oxide (PEO) was investigated at different temperatures in selected organic solvents. With the temperature increase from 25 to 100 °C, there are no changes of solubility of these six ILs (b_1_, b_2_, b_3_, c_1_, c_2_, c_3_) either in nonpolar organic solvents such as anisole and petroleum ether, or in polar solvents such as methanol and ethanol (Table 2). However, the solubility of c_n_ decreases with the increase of temperature in 1,2-dimethoxyethane. Moreover, it can be inferred that the phase transformation temperature (PTT) of PF_6_^−^ based ILs (c_1_, c_2_, c_3_) in 1,2-dimethoxyethane would decrease with the increasing chain length of PEO.

### 2.2. Phase Transformation Temperature of c_2_/1,2-Dimethoxyethane Biphasic Systems

As shown in Table 3, the mixture of [(CH_3_)_2_N(C_2_H_5_)(CH_2_CH_2_O)_2_H][PF_6_] (c_2_) and 1,2-dimethoxyethane show a “homogeneous” liquid phase at 20 °C. c_2_ was precipitated from 1,2-dimethoxyethane (*v*/*v*, 1:1) with the increase of the system temperature to 57 °C, leading to the system separation into two phases. The c_2_ in 1,2-dimethoxyethane possesses the thermosensitive function of “mono-phase under low temperature, separation of the system into two phases under high temperature”. Moreover, the phase transformation temperature (PTT) of the biphasic system decreases with the volume of 1,2-dimethoxyethane increase. The PTT of the biphasic system decreases to 26 °C when the volume ratio of 1,2-dimethoxyethane to water is up to 4:1.

### 2.3. Asymmetric Reduction of Ethyl 2-oxo-4-phenylbutyrate (EOPB) in Thermosensitive Ionic Liquids–Solvent Biphasic System (TIBS)

**Figure 1:**
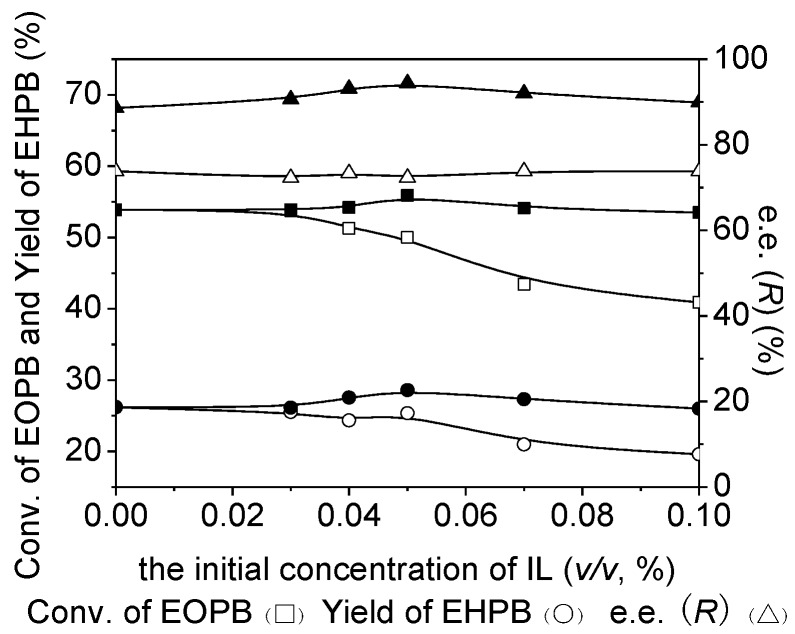
Comparison of biocompatibility of c_2_ and [BMIM][PF_6_] as an additive for the reaction in the benzene-water biphasic system. An amount of 2.00 g baker’s yeast, 0.0825 g EOPB, 20.0 mL benzene, 10.0 mL water, 30 °C, 200 rpm, 5 h. Solid point—c_2_; hollow point—[BMIM][PF_6_].

ILs as (co-)solvents can modulate enzymatic activity by improving substrate specificity, affinity, and enantioselectivity, increasing substrate solubility, and therefore they can be employed to enzymatic reactions. The modulation of activity is caused by a complex combination of effects, differing for each reaction system. The enzyme activity, stability, and enantioselectivity in the ILs media is closely associated with variants of the molecular structure of the ion pairs and enzymes [46,47]. The biocompatibility of c_2_ should be assessed before application as a bioreaction medium. To evaluate the effectiveness of c_2_ on the asymmetric reduction of ethyl 2-oxo-4-phenylbutyrate (EOPB) with baker’s yeast, the conversion ratio (conv.) of EOPB, yield of ethyl 2-hydroxy-4-phenylbutyrate (EHPB), and enantiomeric excesses of ethyl *(R)*-EHPB (e.e. (*R*)) were measured in the presence of a various initial concentration of c_2_ (≤0.1%, *v/v*). As shown in Figure 1, at the initial concentration of 0.05% c_2_ in the benzene-water biphasic system, the e.e. of *(R)*-EHPB increases to 94.4% and the conversion of EOPB and yield of EHPB reach to the top value. Compared with 1-butyl-3-methylimidazolium hexafluorophosphate ([BMIM][PF_6_]), c_2_ is a more biocompatible alternative to the biocatalytic reaction at the same conditions. With the increase of the initial concentration of c_2_ from 0.03% to 0.10% (*v/v*), the e.e. of *(R)*-EHPB, conversion of EOPB, and yield of EHPB show no significant changes (*p* > 0.05). Compared with a biphasic system containing [BMIM][PF_6_], the e.e. of *(R)*-EHPB, conversion of EOPB, and yield of EHPB is significantly higher (*p* < 0.05) in the biphasic system containing c_2_ with initial concentration from 0.04% to 0.10%. Of note, is how the combination of quaternary ammonium cation with multiple ether and/or hydroxyl groups improves the enzymatically synthetic activity for ILs based on the [PF_6_] anion, in agreement with previous results obtained for CAL-B-catalyzing the synthesis with the design of a lipase-friendly solvent that also enables the dissolution of challenging substrates, such as sugars, fatty acids, and triglycerides [43,44,45].

**Figure 2:**
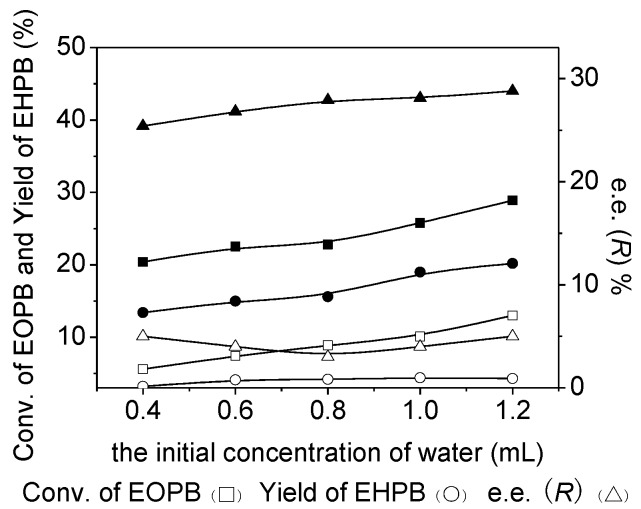
Effect of the initial concentration of water on the reaction by baker’s yeast. An amount of 3.00 g baker’s yeast, 0.165 g EOPB, 5.0 mL c_2_, 18.0 mL 1,2-dimethoxyethane, 30 °C, 24 h. Solid point—in the c_2_/1,2-dimethoxyethane biphasic system; hollow point—in 1,2-dimethoxyethane.

The phase transformation temperature (PTT) of the c_2_/1,2-dimethoxyethane biphasic system (*v/v*, 5:18) is about 33 °C. Therefore, the asymmetric reduction of EOPB was performed at 30 °C, for ensuring that the bioreaction takes place in a “homogeneous” liquid phase. At the end of each reduction run, the system temperature was increased upon to 33 °C and c_2_ was precipitated from 1,2-dimethoxyethane. As shown in Figure 2, the e.e. of *(R)*-EHPB was enhanced about 25~30% and the conversion of EOPB and yield of EHPB were significantly higher (*p* < 0.05) in the c_2_/1,2-dimethoxyethane (*v/v*, 5:18) biphasic system, compared with the reduction in 1,2-dimethoxyethane. Obviously, the existence of c_2_/1,2-dimethoxyethane with the temperature-dependent phase separation property provides fundamental support of the reaction-separation coupling process (RSCP). Moreover, the biocatalyst in the ILs phase can be separated and recycled by a simple phase separation.

**Figure 3:**
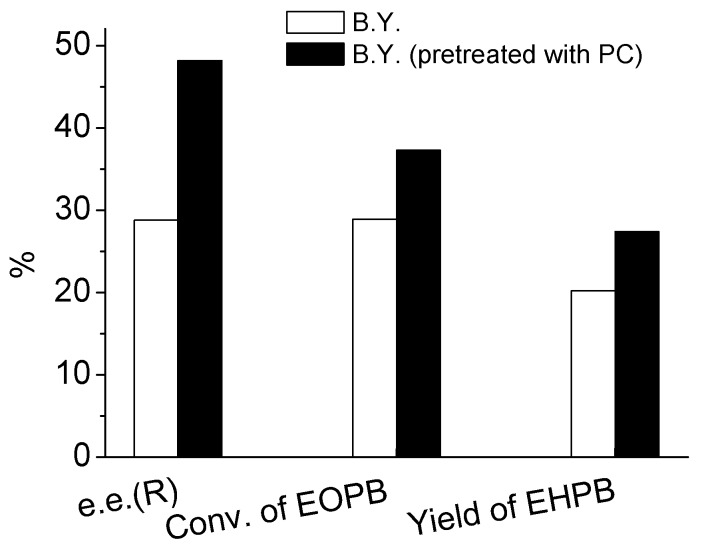
The reduction of ethyl 2-oxo-4-phenylbutyrate (EOPB) catalyzed by baker’s yeast pretreated with alpha-phenacyl chloride (PC) in the thermosensitive c_2_/1,2-dimethoxyethane biphasic system. An amount of 3.00 g pretreated baker’s yeast, 0.165 g EOPB, 1.2 mL H_2_O, 5.0 mL c_2_, 18.0 mL 1,2-dimethoxyethane, 30 °C, 24 h.

Baker’s yeast pretreated by PC was employed to reduce 2-oxo-4-phenylbutyrate (EOPB) in the thermosensitive c_2_/1,2-dimethoxyethane biphasic system (*v/v*, 5:18), compared with the reduction using baker’s yeast without pretreatment by PC at the same conditions (Figure 3). The reduction activity of baker’s yeast and the enantioselectivity of the reduction were improved by employing the baker’s yeast pretreated with alpha-phenacyl chloride (PC) [48]. With the introduction of pretreated baker’s yeast to the reduction, the enantioselectivity of the reduction of EOPB was improved and shifted towards forming the desired configuration (48.3%, e.e. *(R)*-EHPB). Moreover, the conversion ratio (conv.) of EOPB and the yield of EHPB were significantly increased by 9%, respectively, compared with the use of unpretreated baker’s yeast.

## 3. Materials and Methods

### 3.1. Materials

Anisole (CH_3_OC_6_H_5_, 99.7%), petroleum ether (ACS reagent), p-xylene (C_6_H_4_(CH_3_)_2_), ≥99.7%), diethyl ether ((C_2_H_5_)_2_O ACS reagent), methanol (CH_3_OH, ≥99.9%), ethanol (CH_3_CH_2_OH, ≥99.9%), isopropanol ((CH_3_)_2_CHOH, ≥99.7%), 2-methoxyethanol (CH_3_OCH_2_CH_2_OH, ≥99.9%), 1,2-dimethoxyethane (CH_3_OCH_2_CH_2_OCH_3_, 99.9%), nonane (CH_3_(CH_2_)_7_CH_3_, ≥99%), benzene (C_6_H_6_, ≥99.7%), acetone (CH_3_COCH_3_, ≥99.9%), anhydrous sodium acetate (CH_3_COONa, >99%), ethylene oxide (EO, C_2_H_4_O ≥99.5%), *N*,*N*-dimethylethanolamine (DMEA, (CH_3_)_2_NCH_2_CH_2_OH, a_1_), tetrahydrofuran (THF, (CH_2_)_4_O, ≥99.5%), diethyl sulfate (C_4_H_10_O_4_S, 98%), potassium hexafluorophosphate (KPF_6_, ≥99%), diethyl ether anhydrous (C_4_H_10_O, ≥99%), potassium bromide (KBr, FT-IR grade) were purchased from Sigma-Aldrich (Shanghai, China). Unless otherwise stated, all other reagents used were of analytical grade. Ultrapure water produced by an Aquapro purification system (18.2 MΩ cm, Aquapro International Co., Ltd., Dover, DE, USA) was used throughout the experiments.

### 3.2. Preparation of the Ionic Liquid Precursors (a_2_–a_4_)

**Figure 4:**
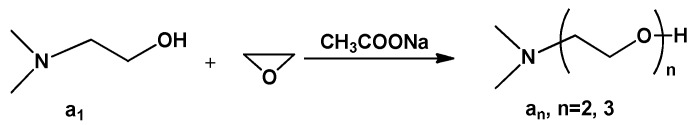
The synthesis route to a_n._

An amount of 100 mL N, N-dimethylethanolamine (a_1_), and 0.4 g anhydrous sodium acetate (CH_3_COONa) were added to a 500 mL stainless-steel high-pressure reactor fitted with temperature controlling apparatus and a mechanical stirrer. The reactor is purged three times with N_2_. The temperature of the reactor which maintained the mixture was raised to 80 °C, and kept at that temperature as 80 mL ethylene oxide (EO) was gradually introduced to the stirred reaction mixture under the pressure below 0.1 MPa. Then, the reaction was continued for 30 min under 0.2 MPa. The ethoxylation products were separated and purified by distillation (75~142 °C) to give 43.37 g of a_2_ (collected at 75~77 °C, 97% purity degree, GC), 38.38 g of a_3_ (collected at 105~107 °C, 96% purity degree, GC), and 31.50 g of a_4_ (collected at 140~142 °C, 94% purity degree, GC). Distillation conditions: Ø 15 × 350 mm packed column, high-performance stainless-steel triangular wire spring fillers, 200 mm filling height, reflux ratio R > 10, 1 mmHg vacuum degree. The ethoxylation procedure is shown in Figure 4.

The purified ethoxylation products were confirmed by a nuclear magnetic resonance spectrometer (NMR, Bruker Avance III, Billerica, USA), a gas chromatography (GC, Agilent 6890, Santa Clara, USA), an electron spray ionization mass spectrometry (ESI-MS, Agilent 6220, Santa Clara, USA), and a Fourier transform infrared spectrometer (FT-IR, Nicolet iS5, Thermo Fisher, USA). ESI-MS: *m/z* 134.4 [a_2_ + H]^+^, 178.4 [a_3_ + H]^+^, 222.4 [a_4_ + H]^+^; *m/z* 200.3 [a_3_ + Na]^+^, 244.4 [a_4_ + Na]^+^. ^1^H-NMR (250 mHz, D_2_O) of a_2_: *δ* = 2.11 (6H, 2×C*H_3_*), *δ* = 2.45 (2H, C*H_2_*CH_2_O), *δ* = 3.52 (4H, C*H_2_*C*H_2_*OH), *δ* = 3.60 (2H, CH_2_C*H_2_*O).^1^H-NMR (250 mHz, D_2_O) of a_3_: *δ* = 2.23 (6H, 2×C*H_3_*), *δ* = 2.49 (2H, C*H_2_*CH_2_O), *δ* = 3.47–3.65 (12H, C*H_2_*C*H_2_*O). ^1^H-NMR (250 mHz, D_2_O) of a_4_: *δ* = 2.18 (6H, 2×C*H_3_*), *δ* = 2.44 (2H, C*H_2_*CH_2_O), *δ* = 3.46–3.62 (16H, C*H_2_*C*H_2_*O).

**Figure 5:**
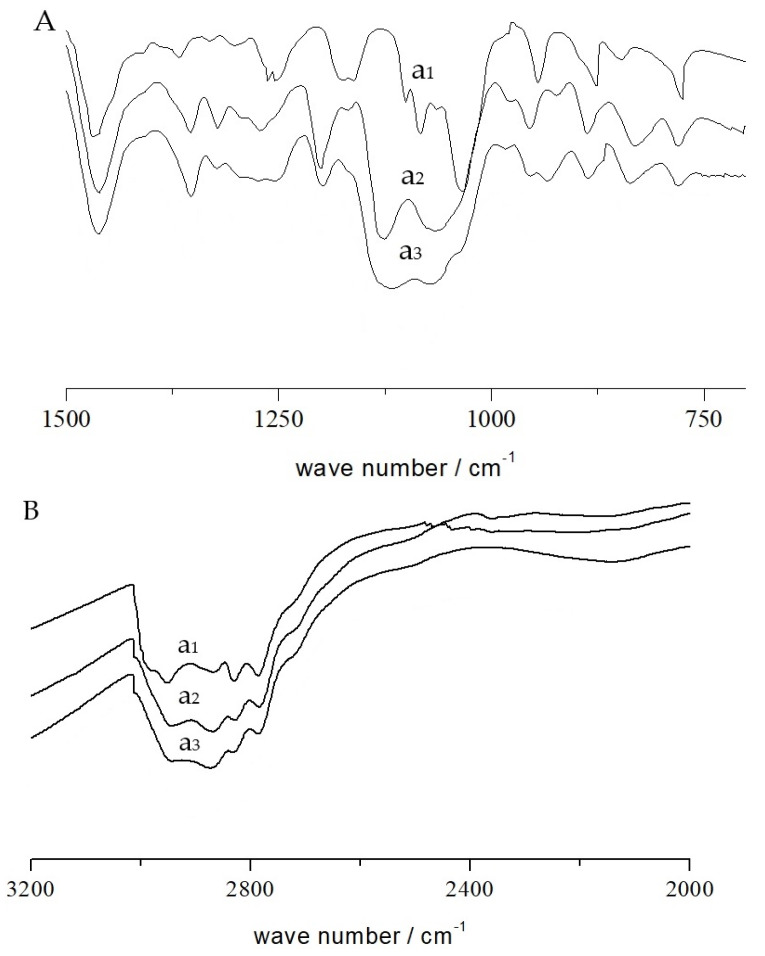
Fourier transform infrared (FT-IR) spectra of *N*,*N*-dimethylethanolamine (DMEA, a_1_), a_2_ ((CH_3_)_2_N(CH_2_CH_2_O)_2_H), and a_3_ ((CH_3_)_2_N(CH_2_CH_2_O)_3_H). (**A**) FT-IR spectra in the wavenumber range from 1500~750 cm^−^^1^; (**B**) FT-IR spectra in the wavenumber range from 3200~2000 cm^−1.^

For the FT-IR spectra measurements, the diffuse reflectance technique was utilized in the range from 4000 to 400 cm^−1^. The samples were ground with a FT-IR grade potassium bromide (KBr) powder and then pressed into 1 mm pellets. The strong absorbance at 1120 cm^−1^ confirmed the formation of ether linkage (–C-O-C–). It is also noticed that the relative peak intensity and peak width of this band were enhanced with the increase of polyether chain length in the molecular structure of EO-derivatives, compared with a_1_ (Figure 5A). Additionally, the evidence of the stronger absorbance at 2960~2825 cm^−1^ for the C–H stretching feature (of –CH_2_, –CH_3_) implied the successful introduction of the poly (EO) chains (Figure 5B).

From the above results, it can be seen that the poly (EO) chains were connected to the OH group from DMEA. The difference between DMEA (a_1_) and its EO-derivatives (a_2_, a_3_ a_4_) is the difference in the polyether chain length.

### 3.3. Preparation of the Ionic Liquid (b_1_~b_3_, c_1_~c_3_)

The synthetic routes to b_1_~b_4_, c_1_~c_3_ are shown in Scheme 1. Products were confirmed by a nuclear magnetic resonance spectrometer (NMR, Bruker Avance III, Billerica, USA), a gas chromatography (GC, Agilent 6890, Santa Clara, USA), and a Fourier transform infrared spectrometer (FT-IR, Nicolet iS5, Thermo Fisher, USA).

b_1_: 18.0 g a_1_ (0.2 mol) and 100 mL tetrahydrofuran (THF) were placed in a 500 mL three-mouth flask. The reactor was purged three times with N_2_. An amount of 30.9 g diethyl sulfate (0.2 mol) was dropped slowly into the flask at 20 °C. The reaction was carried out under reflux for 48 h. The organic solvent was removed under a vacuum and the product was dried under a vacuum (4 mmHg, 60 °C) for 12 h to a viscous transparent oil and weighed (47.81 g, 98.3% yield). FT-IR (KBr, cm^−1^): 3405 ν (O–H); 2907, 2980 ν (–CH_3_), ν (–CH_2_); 1481 δ as (CH_3_), δ (CH_2_); 1224 ν as (OSO_3_^−^); 1068, 1017 ν (N–CH_3_), ν (N–CH_2_); 918 ν as (C–O–S).

The synthesis methods of b_2_, b_3_, and b_4_ are similar to those of b_1_. b_2_: 7.3 g a_2_, 50 mL tetrahydrofuran (THF), 8.45 g diethyl sulfate (54.8 mmol). The product was dried under a vacuum (4 mmHg, 60 °C) for 12 h and weighed (14.14 g, 89.8% yield). b_3_: 17.7 g a_3_, 50 mL THF, 15.4 g diethyl sulfate (100 mmol). The product was dried under a vacuum (4 mmHg, 60 °C) for 10 h and weighed (29.65 g, 89.5% yield). b_4_: 12.5 g a_3_, 50 mL THF, 10.9 g diethyl sulfate (70.7 mmol). The product was dried under a vacuum (4 mmHg, 60 °C) for 10 h and weighed (19.46 g, 83.2% yield). ^1^H-NMR (250 mHz, D_2_O) of b_2_: δ = 1.15 (3H, CH_2_CH_3_), δ = 2.88 (6H, 2×CH_3_), δ = 3.23 (4H, CH_2_CH_2_OH), δ = 3.32 (2H, CH_2_CH_3_), δ = 3.43 (2H, CH_2_CH_2_O), δ = 3.50 (2H, CH_2_CH_2_O). ^1^H-NMR (250 mHz, D_2_O) of b_3_: δ = 1.15 (3H, CH_2_CH_3_), δ = 3.19 (6H, 2×CH_3_), δ = 3.22–3.69 (14H, CH_2_CH_3_, CH_2_CH_2_O), δ = 3.87 (2H, CH_2_CH_2_O), δ = 3.93 (2H, CH_2_CH_2_O).

c_1_: 10.11 g b_1_, 50 mL tetrahydrofuran (THF), and 7.65 g potassium hexafluorophosphate were added to a 100 mL three-mouth flask fitted. The mixture was stirred for 48 h under a nitrogen atmosphere at 20 °C. After filtration, the solvent was removed under a vacuum and the product was dried under a vacuum (4 mmHg, 80 °C) for 12 h to give a viscous transparent oil. IR (KBr, cm^−1^): 3602 ν (O–H); 3359 ν (O–H); 2913, 2989, 3062 ν (–CH_3_), ν (–CH_2_); 1484 δ as (–CH_3_); 1473 δ (–CH_2_); 841 ν (P–F); 558 δ (P–F). The synthesis methods of c_2_ and c_3_ were similar to those of c_1_. c_2_: 14.14 g b_2_, 50 mL THF, and 10.15 g potassium hexafluorophosphate. ^1^H-NMR (250 mHz, D_2_O) of c_2_: δ = 1.17 (3H, CH_2_CH_3_), δ = 2.92 (6H, 2×CH_3_), δ = 3.27 (4H, CH_2_CH_2_OH), δ = 3.36 (2H, CH_2_CH_3_), δ = 3.45 (2H, CH_2_CH_2_O), δ = 3.54 (2H, CH_2_CH_2_O). c_3_: 14.51 g b_3_, 60 mL THF, and 8.07 g potassium hexafluorophosphate. ^1^H-NMR (250 mHz, D_2_O) of c_3_: δ = 1.17 (3H, CH_2_CH_3_), δ = 3.21 (6H, 2×CH_3_), δ = 3.27–3.71 (14H, CH_2_CH_3_, CH_2_CH_2_O), δ = 3.89 (2 H, CH_2_CH_2_O), δ = 4.01 (2H, CH_2_CH_2_O).

### 3.4. Bioreduction

An amount of 0.165 g ethyl 2-oxo-4-phenylbutyrate (EOPB), 1.2 mL H_2_O, 5 mL c_2_, and 18 mL 1,2-dimethoxyethane were well mixed. Then, the reduction was proceeded at 30 °C with stirring at 200 rpm for 24 h. At the end of the reduction run, the temperature was increased to 33 °C, while the reaction system was divided into two phases. The products in the organic solvent were collected and analyzed by GC. The biocatalyst in the ILs phase can be separated and recycled by a simple phase separation. The enantiomeric excesses of (*R*)-EHPB (e.e.(R)) was calculated according to Equation (1):

e.e.(*R*) = (*(R)*-EHPB-*(S)*-EHPB)/(*(R)*-EHPB + *(S)*-EHPB) × 100%, (1)

### 3.5. The Pretreatment of Baker’s Yeast with Alpha-Phenacyl Chloride

An amount of 0.4 g alpha-phenacyl chloride (PC), 50 mL diethyl ether anhydrous, 1.5 mL ultrapure water, and 2.5 g of dry baker’s yeast were agitated at 30 °C, 200 rpm for 2 h. Subsequently, the pretreated baker’s yeast was separated by centrifugation at 4 °C. The residue of PC in yeasts was removed by washing many times with diethyl ether anhydrous until it could not be detected in the final liquid by GC. The pretreated baker’s yeast was obtained after drying under a vacuum and stored at 4 °C.

### 3.6. Statistical Analysis

All analyses were carried out in triplicate. The values were described as the means ± standard deviations. The data were subjected to the analysis of variance (ANOVA) by the Origin program (Origin 9.0). The significance was defined at the 95% confidence level (*p* < 0.05).

## 4. Conclusions

A new thermosensitive ionic liquids–solvent biphasic system (TIBS) has been built with c_2_ and 1,2-dimethoxyethane, which possesses the thermoregulated function of “mono-phase under low temperature, separation of the system into two phases under high temperature”. The phase transformation temperature (PTT) of mixed c_2_/1,2-dimethoxyethane (*v/v*, 5:18) was about 33 °C. This new TIBS was used to the reaction-separation coupling process (RSCP) for the asymmetric reduction of EOPB with baker’s yeast. The enantiomeric excesses of ethyl *(R)*-EHPB were improved about 25~30% and the yield of EHPB was increased 35% in the thermosensitive biphasic system, compared with that in 1,2-dimethoxyethane. With the pretreated by PC, the reduction activity of baker’s yeast and the enantioselectivity of the reduction were improved obviously, shown as the e.e. (*R*) reached to 48.3% with the conversion of EOPB and the yield of EHPB significantly increasing to 9%, respectively. In addition, the baker’s yeast can be separated and recycled with the biocatalyst activity from the ionic liquid by a single phase separation. It is expected that the new TIBS established in this study could provide many future opportunities in biocatalysis.

## Figures and Tables

**Table 1:** Solubilities* of the ionic liquids in solvents at 20 °C.

Solvent	b_1_	c_1_	b_2_	c_2_	b_3_	c_3_
water	s	s	s	s	s	s
anisole	i	i	i	i	i	i
petroleum ether	i	i	i	i	i	i
p-xylene	i	i	i	i	i	i
diethyl ether	i	i	i	i	i	i
methanol	s	s	s	s	s	s
ethanol	s	s	s	s	s	s
isopropanol	i	i	i	i	i	i
2-methoxyethanol	s	s	s	s	s	s
1,2-dimethoxyethane	s	s	i	s	i	i
nonane	i	i	i	i	i	i
benzene	i	i	i	i	i	i
acetone	i	i	i	i	i	s

*s: Solution, i: Insolubilization. b_1_: [(CH_3_)_2_N(C_2_H_5_)CH_2_CH_2_OH][C_2_H_5_SO_4_], c_1_: [(CH_3_)_2_N(C_2_H_5_)CH_2_CH_2_OH][PF_6_], b_2_: [(CH_3_)_2_N(C_2_H_5_)(CH_2_CH_2_O)_2_H][C_2_H_5_SO_4_], c_2_: [(CH_3_)_2_N(C_2_H_5_)(CH_2_CH_2_O)_2_H][PF_6_], b_3_: [(CH_3_)_2_N(C_2_H_5_)(CH_2_CH_2_O)_3_H][C_2_H_5_SO_4_], c_3_: [(CH_3_)_2_N(C_2_H_5_)(CH_2_CH_2_O)_3_H][PF_6_].

**Table 2:** Solubilities of ionic liquids (ILs) modified with different chain lengths of polyethylene oxide in organic solvents under different temperatures.

**Solvent**	**b_1_ (°C)**			**b_2_ (°C)**			**b_3_ (°C)**		
	**25**	**50**	**100**	**25**	**50**	**100**	**25**	**50**	**100**
anisole	i	i	i	i	i	i	i	i	i
petroleum ether	i	i	i	i	i	i	i	i	i
2-methoxyethanol	s	s	s	s	s	s	s	s	s
1,2-dimethoxyethane	s	s	s	i	i	i	i	i	i
benzene	i	i	i	i	i	i	i	i	i
acetone	i	i	i	i	i	i	i	i	i
ethanol	s	s	s	s	s	s	s	s	s
methanol	s	s	s	s	s	s	s	s	s
**Solvent**	**c_1_ (°C)**			**c_2_ (°C)**			**c_3_ (°C)**		
	**25**	**50**	**100**	**25**	**50**	**100**	**25**	**50**	**100**
anisole	i	i	i	i	i	i	i	i	i
petroleum ether	i	i	i	i	i	i	i	i	i
2-methoxyethanol	s	s	s	s	s	s	s	s	s
1,2-dimethoxyethane	s	s	i	s	i	i	i	i	i
isopropanol	-	-	-	i	i	i	i	i	i
ethanol	s	s	s	s	s	s	s	s	s
methanol	s	s	s	s	s	s	s	s	s

*s: Solution, i: Insolubilization. b_1_: [(CH_3_)_2_N(C_2_H_5_)CH_2_CH_2_OH][C_2_H_5_SO_4_], c_1_: [(CH_3_)_2_N(C_2_H_5_)CH_2_CH_2_OH][PF_6_], b_2_: [(CH_3_)_2_N(C_2_H_5_)(CH_2_CH_2_O)_2_H][C_2_H_5_SO_4_], c_2_: [(CH_3_)_2_N(C_2_H_5_)(CH_2_CH_2_O)_2_H][PF_6_], b_3_: [(CH_3_)_2_N(C_2_H_5_)(CH_2_CH_2_O)_3_H][C_2_H_5_SO_4_], c_3_: [(CH_3_)_2_N(C_2_H_5_)(CH_2_CH_2_O)_3_H][PF_6_].

**Table 3:** Effect of 1,2-dimethoxyethane volume on the phase transformation temperature (PTT) of c_2_/1,2-dimethoxyethane biphasic systems.

c_2_ (mL)	1,2-Dimethoxyethane (mL)	20 °C	PTT (°C)
0.1	0.1	homogeneous phase	57.0
0.1	0.2	homogeneous phase	50.0
0.1	0.3	homogeneous phase	47.5
0.1	0.4	homogeneous phase	26.0

## Abbreviation

[BMIM] [PF_6_]	1-butyl-3-methylimidazolium hexafluorophosphate
DMEA	*N*, *N*-dimethylethanolamine
EHPB	ethyl 2-hydroxy-4-phenylbutyrate
e.e. (R)	enantiomeric excesses of ethyl *(R)*-2-hydroxy-4-phenylbutyrate
*(R)*-EHPB	ethyl *(R)*-2-hydroxy-4-phenylbutyrate
EO	ethylene oxide
EOPB	ethyl 2-oxo-4-phenylbutyrate
ESI-MS	electron spray ionization mass spectrometry
FT-IR	Fourier transform infrared spectroscopy
GC	gas chromatography
^1^H-NMR	proton nuclear magnetic resonance
IL	ionic liquid
ILs	ionic liquids
PC	alpha-phenacyl chloride
PEO	polyethylene oxide
PTT	phase transformation temperature
RSCP	reaction-separation coupling process
THF	tetrahydrofuran
TIBS	thermosensitive ionic liquids–solvent biphasic system
